# Assessment right atrial thrombus by real-time three dimensional transthoracic echocardiography in patient with dilated cardiomyopathy

**DOI:** 10.1186/1476-7120-9-12

**Published:** 2011-04-10

**Authors:** Wenjuan Bai, Hui Li, Qing Zhang, Li Rao

**Affiliations:** 1 Echo lab, Division of Cardiology Medicine, West China Hospital of Sichuan University, Chengdu, (610041), China; 2 Division of Cardiology Medicine, West China Hospital of Sichuan University, Chengdu, (610041), China

**Keywords:** Thrombus, Dilated cardiomyopathy, Echocardiography

## Abstract

We report a case of a 52-year-old patient with dilated cardiomyopathy who presented with worsening heart failure. Two-dimensional transthoracic echocardiography and real-time three dimensional transthoracic echocardiography showed severe dilated cardiac chambers, impaired ejection fraction and a mobile right atrial thrombus 2.6 × 1.0 cm in size, traversing the right atrial cavity during the whole cardiac cycle. After one week therapeutic anticoagulation, echocardiography confirmed no evidence of residual thrombus.

## Introduction

Dilated cardiomyopathy (DCM) is a kind of malignant cardiac disorder, which affects 5 in 100,000 adults and 0.57 in 100,000 children [1,2]. It represents a poor long-term prognosis with the mortality rate is 70% to 80% at eight years for most patients who undergo heart failure [3]. Right heart thrombus presents higher mortality [4] with a risk of pulmonary embolization [5], which has been found in patients with DCM rarely. Echocardiography plays a pivotal role in the evaluation of DCM and intracardiac masses. To the best of our knowledge, this is the first case report using combination of two-dimensional echocardiography and real-time three dimensional echocardiography in the assessment of right atrial thrombus happened in DCM.

## Case presentation

**Figure 1:**
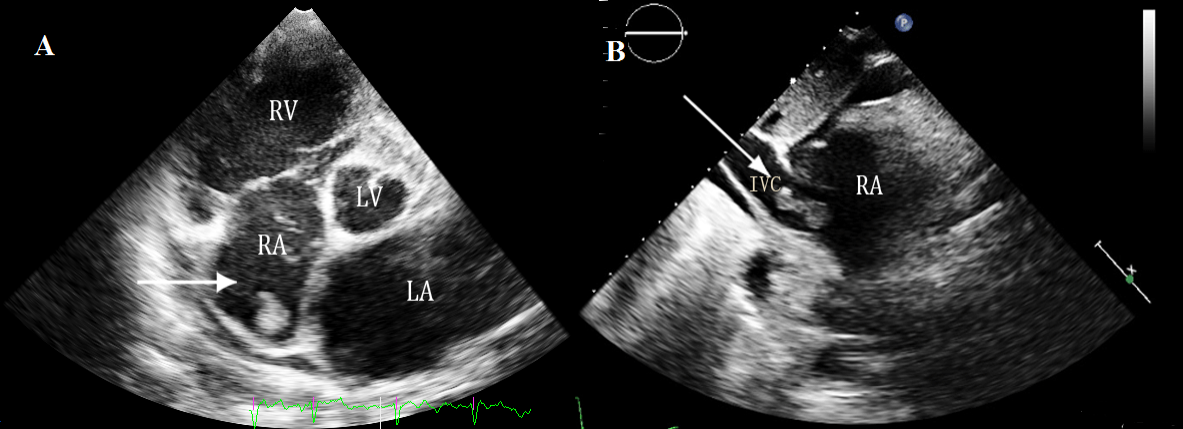
**A mass of right atrium**: A) Parasternal short axis view by 2D TTE, B) Subxyphoid view by 2D TTE.

**Figure 2:**
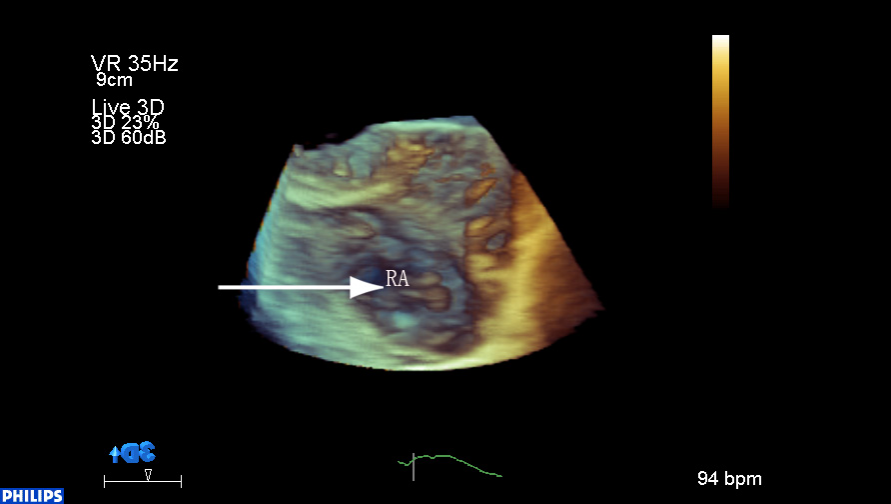
**Mobile mass of right atrium**: RT-3DTEE shows a 2.6 × 1.0 cm homogeneously echogenic mobile mass which floating around the orifice of inferior vena cava and protruding into the right atrial cavity (arrow).

**Figure 3:**
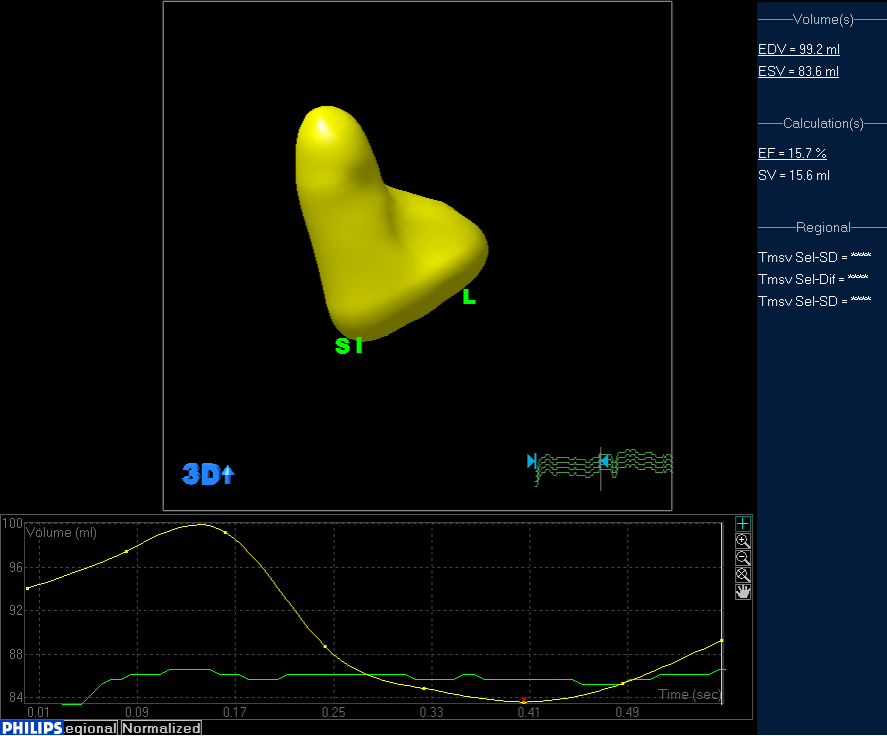
**Right ventricular ejection fraction**: RT-3DTEE measured right ventricular systolic dysfunction with an EF 15.7%.

**Figure 4:**
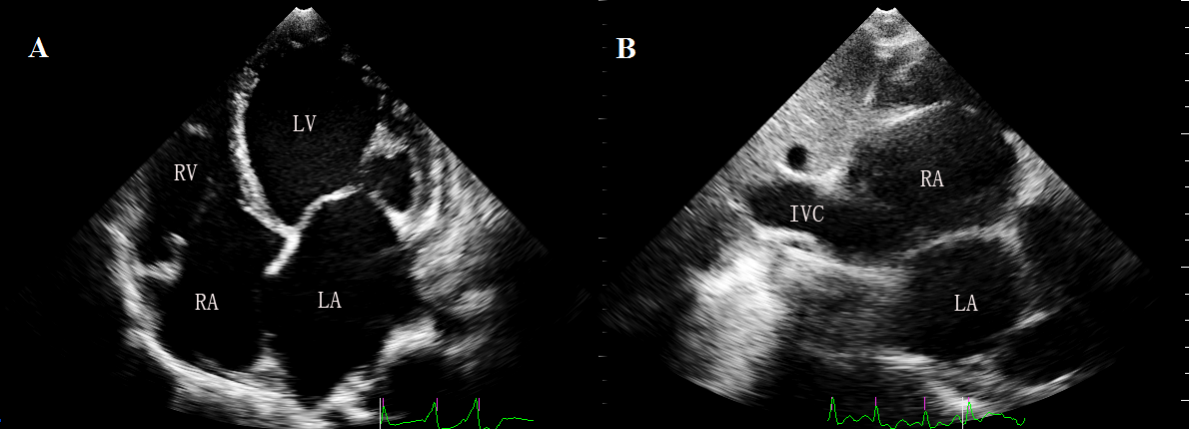
**Follow up after 1 week anticoagulation**: In A) apical 4-chamber view and B) subxyphoid view shows complete disappearance of the thrombi. LV = left ventricle; RA = right atrium; RV = right ventricle, LA = left atrium, IVC = inferior vena cava.

A 52-year-old man (body surface area: 1.3 m^2^) known to have dilated cardiomyopathy (DCM), was admitted for worsening heart failure (NYHA class IV). He presented with a 5-month history of cough, progressive dyspnoea, orthopnea and recurrent upper abdominal pain started from 2 months ago. On examination his vital signs were body temperature 37°C, blood pressure 80/40 mmHg, respiratory rate 24 breaths per minute, and electrocardiogram showed atrial fibrillation with heart rate 50 beats per minute. Physical examination revealed the jugular venous distension, significant tender hepatomegaly and bilateral pitting edema at lower limbs. Laboratory tests showed elevated pro-NT brain natriuretic peptide of 22145 pg/ml (normal 0 to 227 pg/ml) and unremarkable D-dimer. X-ray and computed tomography of the chest demonstrated consolidation of bilateral lower lobes with pleural effusion, while his venous Doppler of lower extremities was normal. Based on his clinical condition, echocardiography was immediately inserted. There were severe dilated cardiac chambers, especially enlargement of the left ventricle (LV) (58 mm/m2) with spherical shape, decreased wall thickness, impaired ejection fraction 22% and severe mitral regurgitation on two-dimensional transthoracic echocardiography (2DTTE). Parasternal short axis and subxyphoid view (Figure 1) showed the mobile right atrial mass highly suspicious of a thrombus traversing the right atrial cavity during the cardiac cycle accompanying with free-floating small parts of the thrombi. Real-time three dimensional transthoracic echocardiography (RT-3DTTE) was performed to further confirm the nature of mass. It showed a highly mobile thrombus, irregular in contour, measured 2.6 × 1.0 cm which floating around the orifice of inferior vena cava and protruding into the right atrial cavity (Figure 2). In addition, RT-3DTTE evaluated right ventricle (RV) systolic dysfunction with ejection fraction 15.7% (Figure 3). He was maintained on digoxin, spironolactone, furosemide, sotalol and dopamine. At the same time therapeutic anticoagulation was started with low-molecular-weight heparin and warfarin. The patient had an uneventful hospital course and one week follow-up echocardiography confirmed adequate removal of the thrombus (Figures 4A and 4B).

## Discussion

DCM is a multifactorial myocardial disease characterized by enlarged left or whole heart chambers and decreased heart function [6]. Symptoms of progressive heart failure are common in patients with dilated cardiomyopathy and endstage disease may predispose to arrhythmia associated with a high risk of thrombus formation which may lead to thromboembolic events [7]. Right heart thrombus happens about 4-18% cases with acute pulmonary embolism [8,9], while rare found in patients with DCM. They may develop within right heart because of atrial fibrillation, hypercoagulable state caused by disease or origin from peripheral venous clots due to bedridden condition that temporarily embolized to the right heart. This type of thrombus presents higher mortality [4] with a risk of potential fragmentation and causing massive pulmonary embolization [5].

Echocardiography is the most important non-invasive comprehensive method in clinical diagnosis of DCM as well as evaluating intracardiac masses or thrombi. In our report, the patient had a right atrial mass with the severity of both ventricle dysfunctions. Although conventional 2DTTE can help to estimate cardiac structure, function and assess the hemodynamic effects, the RT-3DTTE adds valuable benefits as follows: (1) the RT-3DTTE can provide complete information of intracardiac masses[10] including size, shape, consistency, mobility and location that in relation to cardiac anatomical structures.(2) It allows us to differentiate the thrombus from other masses (e.g. cardiac tumors, vegetations) by revealing lobula, stalk, hyperechogenicity or hypoerechogenicity and spatial relationship with adjacent structures, which may help in demonstrating prognostic indications or the response to treatment.(3) In addition, RV dysfunction plays an important role in adverse outcome [11], RT-3DTTE provides a practical approach for quantified the RV function without relying on the geometric assumptions. (4) Patients with DCM usually have distorted LV shape. RT-3DTTE can quantify the LV function without long time breath holding and fully cooperative, also the result is more accurate even like as MRI technology [12]. In our presentation, the combination of 2DTTE and RT-3DTTE may facilitate the diagnosis in evaluating heart function and characterize the nature of thrombus better.

## Consent

Written informed consent was obtained from the relative of the patient for publication of this case report and any accompanying images. A copy of the written consent is available for review by the Editor-in-Chief of this journal.

## Authors' contributions

WB performed the echocardiography examination and drafted the manuscript. HL carried out the quantitative analysis. QZ performed the physical examination and collected the experimental materials. LR conceived of the report. All authors read and approved the final manuscript.

## Competing interests

The authors declare that they have no competing interests.
